# Differential effects of LifeAct-GFP and actin-GFP on cell mechanics assessed using micropipette aspiration

**DOI:** 10.1016/j.jbiomech.2015.12.034

**Published:** 2016-01-25

**Authors:** Kristina Sliogeryte, Stephen D. Thorpe, Zhao Wang, Clare L. Thompson, Nuria Gavara, Martin M. Knight

**Affiliations:** Institute of Bioengineering and School of Engineering and Materials Science, Queen Mary University of London, London, United Kingdom

**Keywords:** Actin cytoskeleton, Micropipette aspiration, Cell mechanics, Bleb, Mesenchymal stem cell

## Abstract

The actin cytoskeleton forms a dynamic structure involved in many fundamental cellular processes including the control of cell morphology, migration and biomechanics. Recently LifeAct-GFP (green fluorescent protein) has been proposed for visualising actin structure and dynamics in live cells as an alternative to actin-GFP which has been shown to affect cell mechanics. Here we compare the two approaches in terms of their effect on cellular mechanical behaviour. Human mesenchymal stem cells (hMSCs) were analysed using micropipette aspiration and the effective cellular equilibrium and instantaneous moduli calculated using the standard linear solid model. We show that LifeAct-GFP provides clearer visualisation of F-actin organisation and dynamics. Furthermore, LifeAct-GFP does not alter effective cellular mechanical properties whereas actin-GFP expression causes an increase in the cell modulus. Interestingly, LifeAct-GFP expression did produce a small (~10%) increase in the percentage of cells exhibiting aspiration-induced membrane bleb formation, whilst actin-GFP expression reduced blebbing. Further studies examined the influence of LifeAct-GFP in other cell types, namely chondrogenically differentiated hMSCs and murine chondrocytes. LifeAct-GFP also had no effect on the moduli of these non-blebbing cells for which mechanical properties are largely dependent on the actin cortex. In conclusion we show that LifeAct-GFP enables clearer visualisation of actin organisation and dynamics without disruption of the biomechanical properties of either the whole cell or the actin cortex. Thus the study provides new evidence supporting the use of LifeAct-GFP rather than actin-GFP for live cell microscopy and the study of cellular mechanobiology.

## 1 Introduction

The actin cytoskeleton plays a key role in many cellular processes such as mechanotransduction (Janmey and Weitz, 2004), motility (Pollard and Cooper, 2009) and differentiation (Titushkin and Cho, 2011). The organisation and dynamic remodelling of cortical actin also influence the structure and biomechanics of cells (Tan et al., 2008, Yourek et al., 2007). The actin cortex is connected to the cell membrane via the family of ezrin, radixin and moesin (ERM) linker proteins (Charras et al., 2006). Mechanical rupture or physiological disassembly of these linker proteins or the underlying actin cytoskeleton results in membrane detachment from the cortex and the formation of a membrane bleb with important consequences for cell biomechanics and migration (Fackler and Grosse, 2008, Sliogeryte et al., 2014). Cell biomechanical properties are therefore associated with actin structure and dynamics and membrane bleb formation, and play a role in dictating the cellular response to the extracellular mechanical environment (Ingber, 2006, Zhelev et al., 1994).

Actin monomers exist in a globular G-actin form that polymerises into fibrous F-actin microfilaments. There is constant turnover between the two states, known as actin treadmilling, such that F-actin is able to form dynamic intracellular structures such as lamellipodia, filopodia, stress fibre bundles and cortical actin. With the increasing interest in understanding actin dynamics and its diverse roles within cell biology, the visualisation of actin in living cells has become an important and powerful technique. Live cell imaging of actin remodelling and dynamics has been widely reported through the transfection of cells with a plasmid expressing actin coupled to a fluorescent protein such as GFP (Endlich et al., 2007). This approach labels both F-and G-actin, which can be useful for assessing the relative dynamics (Engelke et al., 2010) but also reduces the signal to noise ratio when visualising F-actin structures (Lee et al., 2013). Importantly, studies have reported that actin-GFP expression directly influences actin dynamics during cell cytokinesis and migration (Aizawa et al., 1997), cell-matrix adhesion (Feng et al., 2005), and mechanically induced cell deformation (Deibler et al., 2011, Pravincumar et al., 2012).

Alternatively, actin can be labelled through fusion of a fluorescent protein to the actin-binding domain of a known actin binding protein. Such tools include Utrophin (Burkel et al., 2007), F-tractin (Johnson and Schell, 2009) and LifeAct (Riedl et al., 2008), while more recently, a far-red small molecule probe incorporating silicone-rhodamine and an actin binding domain, SiR-actin, has been developed with potential applications in live cell super-resolution microscopy (Lukinavicius et al., 2014). While each of these probes is subject to some bias in cellular distribution when compared to phalloidin, LifeAct provides a balanced choice with good definition of actin structure and no observed side effects (Belin et al., 2014); and remains widely used. Riedl et al. (2008) first described the use of LifeAct tagged to a fluorescent protein as a means of labelling F-actin with reduced artefacts. LifeAct is a peptide consisting of 17-amino-acids comprising the actin-binding domain from yeast actin binding protein 140 (ABP140), which because of its small size and absence from mammalian cells, is ideal for binding F-actin with minimal disruption. Furthermore, no effects on cell migration or polarisation have been observed with its use (Riedl et al., 2008). However, little is known about how LifeAct influences actin dynamics and remodelling during cell deformation. The aim of this paper is to assess the effects of both LifeAct-GFP and actin-GFP on cellular mechanical properties and bleb formation assessed via micropipette aspiration.

## 2 Methods

### 2.1 Cell sources and culturing conditions

Human bone marrow derived mesenchymal stem cells (hMSCs) were purchased from a commercial source (STEMCELL Technologies, Cambridge, UK). For passage culture, cells were seeded at a density of 5×10^3^ cells/cm^2^ and cultured in media consisting of low glucose Dulbecco’s Modified Eagle Media (DMEM; Gibco, Paisley, UK) with 10% foetal bovine serum (FBS), penicillin (100 U/mL)-streptomycin (100 µg/mL; all Sigma-Aldrich, Dorset, UK) and 1 ng/mL fibroblast growth factor-2 (FGF-2; PeproTech, London, UK) at 37 °C and 5% CO_2_ until confluence of 70–80% was reached as previously described (Pattappa et al., 2013). Cells between passages 2 and 8 were used for experiments and were cultured in 24-well plates at an initial density of 5×10^3^ cells/cm^2^ for seven days before transfection.

For chondrogenic differentiation, hMSCs were cultured in medium consisting of high glucose DMEM, (1×) Insulin-Transferrin-Selenium-G supplement (both Gibco), penicillin (100 U/mL)-streptomycin (100 µg/mL), 1 mM sodium pyruvate, 1.5 mg/mL bovine serum albumin (BSA), 40 µg/mL l-proline, 4.7 µg/mL linoleic acid, 50 µg/mL l-ascorbic acid, 100 nM dexamethasone (all Sigma-Aldrich) and 10 ng/mL transforming growth factor-β3 (TGF-β3; PromoKine, Heidelberg, Germany) as described previously (Sliogeryte et al., 2014).

A conditionally immortalised wild-type mouse chondrocyte cell line was also used. In this case, cells were cultured in DMEM (Gibco) supplemented with 10% FBS, penicillin (100 U/mL)-streptomycin (100 μg/mL), and 2.5 mM l-glutamine (all Sigma-Aldrich). Immortalised cells were maintained under permissive conditions at 33 °C, 5% CO_2_ in the presence of 10 nM interferon-γ (IFN-γ; R&D Systems, Abingdon, UK) (Thompson et al., 2014, Wann et al., 2012). Cells were then cultured under non-permissive conditions at 37 °C in the absence of IFN-γ for 3 days followed by seeding in 24 well plates 24 h before experiments.

For micropipette aspiration experiments, all cell types were detached with 0.25% Trypsin/EDTA (Sigma-Aldrich) for 3–5 min, pelleted and suspended in pre-warmed imaging medium consisting of low glucose DMEM (no Phenol Red; Gibco), penicillin (100 U/mL)-streptomycin (100 µg/mL), 10% FBS, 4 mM l-Glutamine and 25 mM HEPES (all Sigma-Aldrich). Following detachment, the cell suspension was incubated in a water bath for 10–15 min prior to micropipette aspiration.

### 2.2 LifeAct-GFP and actin-GFP transfections

For actin-GFP transfection, undifferentiated hMSCs were transfected with a plasmid driving expression of actin-GFP. Prior to transfection cells were cultured in antibiotic free media (low glucose DMEM with 10% FBS) for 30 min to 1 h. Plasmid transfection was performed using Lipofectamine LTX Plus (Invitrogen, Paisley, UK). For 2×10^4^ cells, 0.5 μg of cDNA was used. Cells were cultured for 6 h in transfection media according to the manufacturer’s instructions. Undifferentiated hMSCs, hMSCs differentiated toward the chondrogenic lineage and an immortalised chondrocyte cell line were transfected with an adeno-virus containing LifeAct-TagGFP2 (Ibidi, Martinsried, Germany) at a pre-optimised multiplicity of infection (MOI) according to the manufacturer’s protocol. Two days prior to experimental observation, the reagent was directly added to the cells cultured in monolayer. The cells were incubated for two days at 37 °C, 5% CO_2_. After incubation with either virus or plasmid the media was replaced. Cell viability remained high after introduction of either actin-GFP or LifeAct-GFP to cells. Control cells were cultured in parallel without subjection to transduction or transfection procedures. Prior to micropipette aspiration, cells were treated with trypsin and suspended in imaging media. For imaging of monolayer cells, both groups were seeded and transfected on coverslips.

### 2.3 Visualisation of actin structure in fixed cells

For visualisation of F-actin structure in cell monolayer, cells cultured on cover slips were transfected with LifeAct-GFP or actin-GFP, fixed in 4% paraformaldehyde (PFA) for 10 min, permeabilised for 5 min in 0.5% Triton X-100/phosphate buffered saline (PBS) and stained with Alexa Fluor 555-phalloidin (1:40; Invitrogen) at 25 μl/ml in PBS+0.1% bovine serum albumin (BSA; Sigma-Aldrich) for 20 min. Coverslips with cells were then washed in PBS and mounted with ProLong Gold (Invitrogen).

For visualisation of F-actin structure in rounded cells, the following procedure was performed. Transfected cells with LifeAct-GFP or actin-GFP were detached using trypsin, suspended in imaging media and fixed in 4% PFA for 10 min, followed by permeabilisation in 0.5% Triton X-100/PBS (Sigma-Aldrich) for 5 min prior to staining with Alexa Fluor 555-phalloidin (1:40; Invitrogen) in PBS+0.1% BSA for 20 min. Cells were then washed in PBS and suspended in distilled water. A drop of stained cells in suspension was placed on a coverslip and allowed to dry. Coverslips with cells were mounted using ProLong Gold and imaged using a laser scanning confocal microscope (Leica TCS SP2) with a ×40/1.25 NA oil immersion objective lens. The plane of focus was made to bisect the centre of individual cells.

### 2.4 Micropipette aspiration

The micropipette aspiration system controlled by a peristaltic pump (MCD standard, Ismatec, Cole-Parmer, London, UK) was used as previously described (Pravincumar et al., 2012). The pump was used to provide precise temporal control of aspiration pressure. Micropipettes were made from borosilicate glass capillary tubes (1.0 mm outer diameter and 0.58 mm inner diameter, Narishige, London, UK). The micropipettes were drawn with a programmable Flaming/Brown micropipette puller, (Model P-97, Sutter Instruments Co., Novato, CA, USA). To obtain an inner diameter of 7–8 μm, the micropipettes were fractured on a microforge (MF-900, Narishige) and coated with Sigmacote (Sigma-Aldrich) to prevent cell adhesion. Before starting an experiment, the reservoir, tubing and pump were filled with distilled water taking care to exclude all air bubbles. The micropipettes were filled with imaging media and mounted on a holder controlled by a micromanipulator (Patchman NP2, Eppendorf, Germany). The cell suspension at room temperature was placed in a chamber on the microscope and a tare pressure of 50 Pa was applied to attach an individual cell to the micropipette. The cell was then partially aspirated inside the micropipette by applying a step negative pressure of 0.76 kPa at a rate of 0.38 kPa/s. Brightfield and fluorescence images were captured every 2 s over 3 min using a confocal microscope (Leica, SP2) with a ×63/1.4 NA oil immersion objective lens. Cell elongation into the micropipette was measured from brightfield images using a Matlab routine. Micropipette aspiration was performed within 1 h following cell detachment from monolayer.

### 2.5 Estimation of viscoelastic properties

Viscoelastic parameters such as the equilibrium modulus, the instantaneous modulus and the viscosity of cells were estimated by fitting the theoretical standard linear solid (SLS) model to the obtained aspirated length versus time data using a Matlab routine as described in previous studies (Sato et al., 1990, Theret et al., 1988, Trickey et al., 2000). In this model the cell is assumed to be homogeneous and incompressible with a Poisson’s ratio of 0.5. The model is presented as two parallel connected springs with elastic constants *k*_1_ and *k*_2,_ and a dashpot with viscosity *μ* in series with spring *k*_2_. Applying a negative pressure, the cell elongation into the micropipette is calculated as a function of time, as follows:

(1) $$L\left(t\right)=\frac{\Phi (\eta )R_{p}\Delta p}{\pi E}\times \left[1+\left(\frac{k_{1}}{k_{1}+k_{2}}-1\right)\exp\left(-\frac{t}{\tau }\right)\right]$$

where *L*(*t*) is the aspirated length at time *t*, Δ*P* is applied pressure, *R*_p_ is the inner radius of the micropipette and *Φ*(*η*) is a wall function which in a wide range of experiments was assumed to be 2.0–2.1 (Theret et al., 1988). The cell viscosity can be estimated as follows:

(2) $$\mu =\frac{\tau k_{1}k_{2}}{k_{1}{+k}_{2}}$$

where *τ* is the exponential time constant, and elastic constants *k*_1_ and *k*_2_ are related to the equilibrium (*E*_∞_) and the instantaneous (*E*_0_) moduli as given below:

(3) $$E_{0}=\frac{3}{2}\;(\;k_{1}+\;k_{2});\;E_{\infty }=\;\frac{3}{2}\;k_{1}$$

### 2.6 Statistics

For cell viscoelastic properties the values in the graphs are presented as a population with median values indicated. Statistical analyses were performed using GraphPad Prism (La Jolla, CA, USA). The Mann–Whitney *U* test and Chi-squared test were used to compare datasets with significance indicated by *p*<0.05.

## 3 Results and discussion

**Fig. 1:**
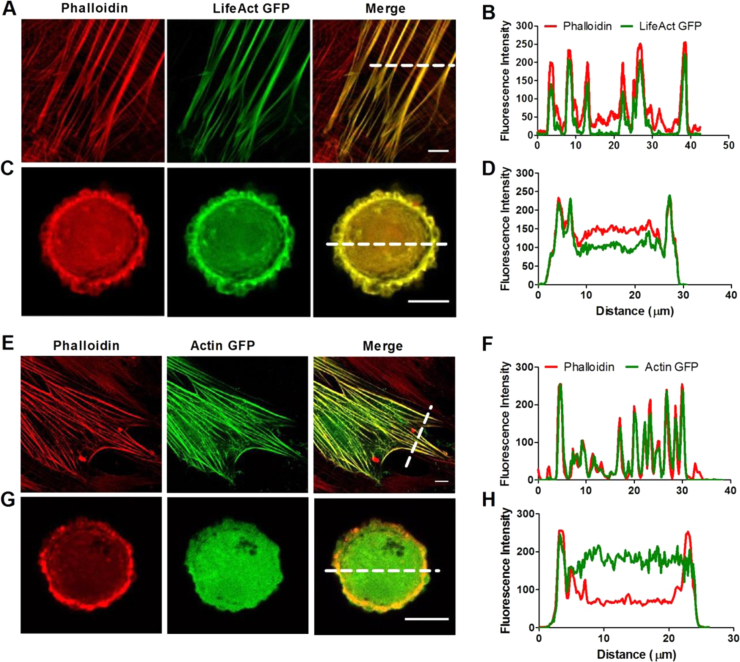
Co-localisation of F-actin labelled with LifeAct-GFP or actin-GFP and Alexa Fluor 555-phalloidin. hMSCs were transfected with LifeAct-GFP or actin-GFP, fixed and counter stained with Alexa Fluor 555-phalloidin. Confocal images of representative cells expressing LifeAct-GFP (A and C) or actin-GFP (E and G) and imaged in monolayer (A and E) or suspension (C and G) culture. White dotted lines indicate the position of fluorescent intensity profiles across the actin stress fibres in monolayer (B and F) or cortical actin in suspension (D and H). Scale bars represent 10 µm.

Actin in hMSCs was labelled with either LifeAct-GFP or actin-GFP. Initial studies used laser scanning confocal microscopy to compare the co-localisation of LifeAct-GFP or actin-GFP with F-actin labelled using Alexa Fluor 555 conjugated phalloidin (Fig. 1). Co-localisation was investigated in both rounded cells in suspension and in monolayer cultured cells which possess different actin organisations. For cells in monolayer, both LifeAct-GFP and actin-GFP accurately labelled F-actin stress fibre bundles as shown by the excellent co-localisation with Alexa Fluor 555-phalloidin (Fig. 1A, B, E and F). Similarly, for rounded cells cortical F-actin was successfully labelled by both LifeAct-GFP and actin-GFP, although the latter also presented greater cytoplasmic labelling intensity, probably G-actin, which reduced the clarity of the F-actin image (Fig. 1C, D, G and H). However in relation to photobleaching experiments not investigated herein, it has been shown that the low affinity of LifeAct to F-actin does limit its use when compared to labelled actin subunits (Riedl et al., 2008).

**Fig. 2:**
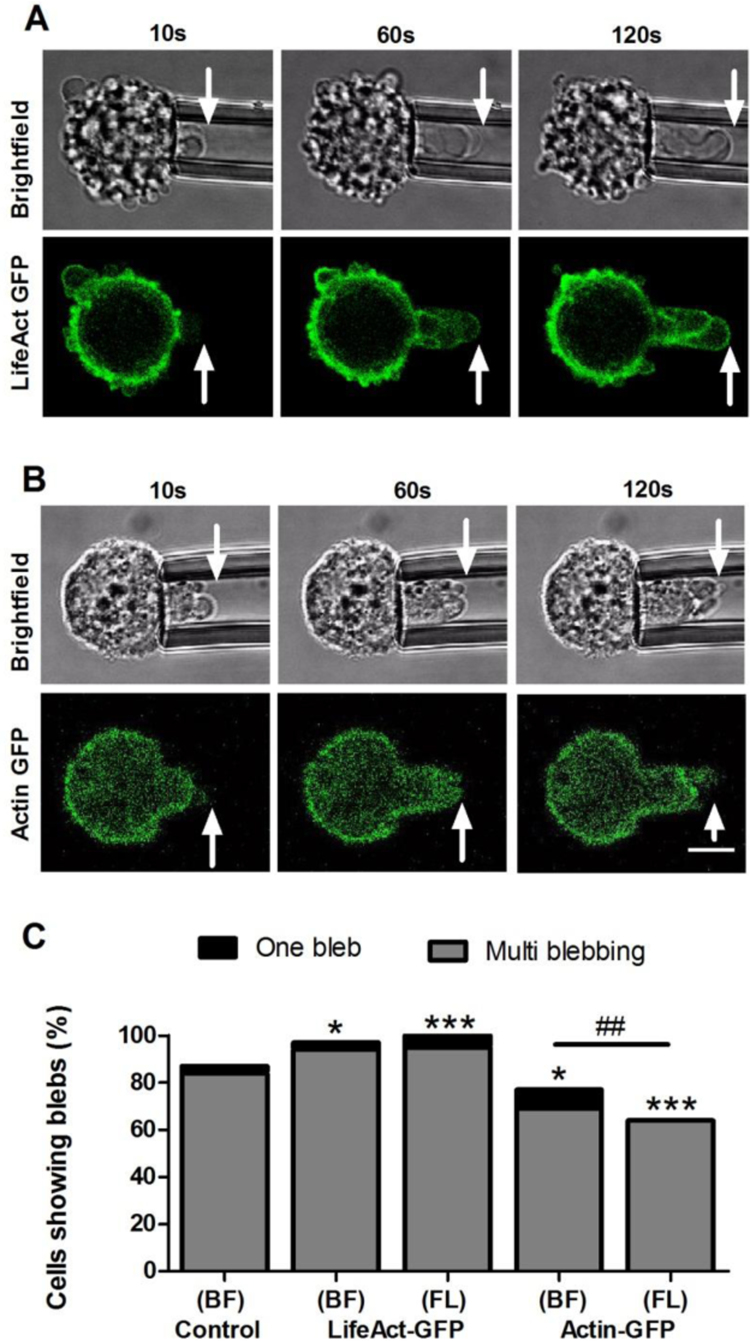
LifeAct-GFP provides clear visualisation of actin deformation, remodelling and bleb formation compared to actin-GFP. Brightfield and confocal fluorescence images of actin dynamics during micropipette aspiration for two representative hMSCs expressing LifeAct-GFP (A) or actin-GFP (B). Images taken at 10, 60 and 120 s after the application of a 0.76 kPa aspiration pressure. White arrows indicate the leading edge of the cell aspirated inside the micropipette based on brightfield images. Scale bar represents 10 µm. (C) Histograms showing the percentage of cells exhibiting membrane blebs. Data presented from two independent experiments, *n*=67 (control), *n*=38 (LifeAct-GFP) and *n*=39 (Actin-GFP). Statistical analyses using the Chi-squared test are indicated relative to non-transfected (BF) control (*: *p*<0.05, ***: *p*<0.001) and between percentages estimated from brightfield (BF) and fluorescence (FL) microscopy (##: *p*<0.01).

Micropipette aspiration was then used in combination with confocal microscopy to visualise fluorescently-labelled actin dynamics as cells were deformed by suction into a micropipette. A step negative pressure of 0.76 kPa was applied at a rate of 0.38 kPa/s and held for 180 s allowing visualisation of the gradual viscoelastic deformation of the cell inside the micropipette (Fig. 2A and B). In approximately 85% of non-transfected control cells, deformation into the micropipette was associated with the formation of a membrane bleb which was visible in brightfield images as a transparent extension (see Supplementary movie S1). About 94% of these bleb forming cells exhibited the formation of multiple successive blebs (multi-blebbing) at the leading edge within the micropipette. Cells transfected with LifeAct-GFP enabled visualisation of actin dynamics during deformation and bleb formation. Micropipette aspiration of hMSCs resulted in initial deformation of the actin cortex followed by membrane-cortex detachment and bleb formation. This was followed by the formation of a new actin cortex at the leading edge of the bleb (Fig. 2A). The process was then repeated with the formation of a new bleb and development of a new actin cortex (multi-blebbing; see Supplementary Movie S2, Movie S3, Movie S4, Movie S5). Cortical actin organisation and remodelling during micropipette aspiration was less clearly defined in cells transfected with actin-GFP, making it more difficult to clearly identify bleb formation from the fluorescence images (Fig. 2B, Supplementary Movie S6, Movie S7). This led to a significant reduction in the percentage of bleb forming cells identified from fluorescent images compared to brightfield images (*p*<0.01; Fig. 2C). This difference between brightfield and fluorescent imaging was not observed in cells transfected with LifeAct-GFP. LifeAct-GFP expression slightly increased the percentage of cells exhibiting membrane blebs compared to non-transfected cells (*p*<0.05; Fig. 2C). Conversely, cells transfected with actin-GFP exhibited a significant reduction in bleb formation. This decrease is probably due to increased actin density as a result of actin overexpression which increases the physical interaction between the cell membrane and actin cortex as previously shown for stem cell differentiation towards the osteogenic (Titushkin and Cho, 2011) and chondrogenic lineages (Sliogeryte et al., 2014). The increase in bleb incidence with LifeAct-GFP may be caused by concealment of membrane-actin linker protein binding sites on F-actin by LifeAct-GFP, leading to a reduction in the number of links between the plasma membrane and actin cortex, allowing for easier detachment of the membrane from the cortex.

**Fig. 3:**
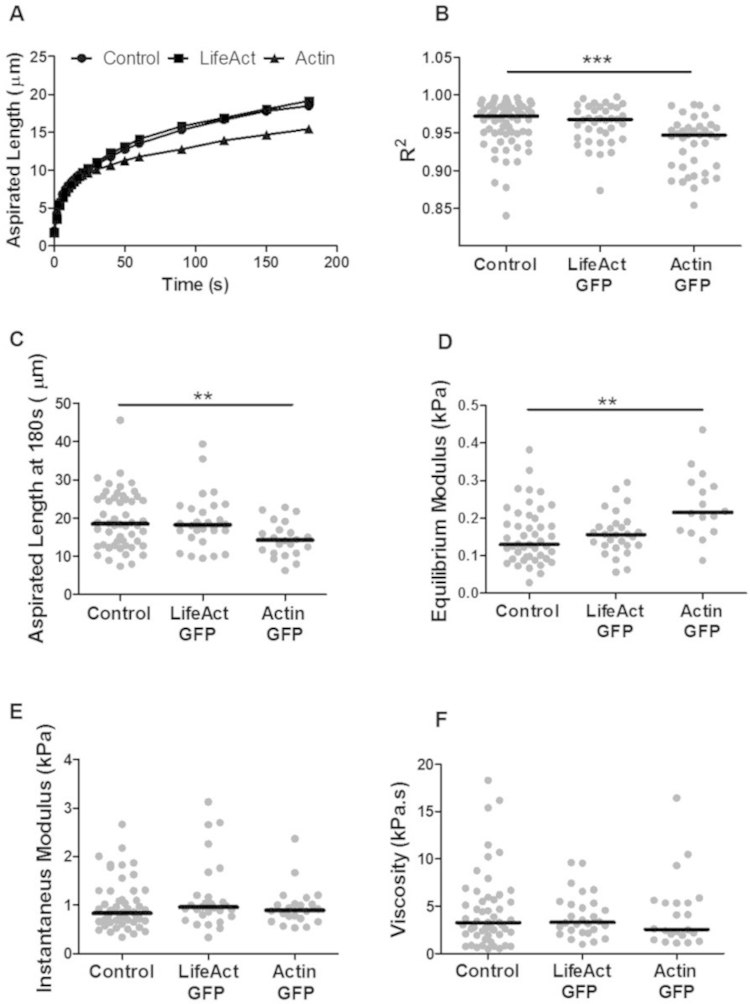
LifeAct-GFP does not interfere with cell biomechanical properties. (A) Plots of median aspirated length versus time for control, LifeAct-GFP and actin-GFP hMSCs. hMSCs transfected with actin-GFP exhibit a shorter aspirated length compared to control cells. Corresponding plots showing (B) fitting parameter *R*^2^, (C) aspirated length at *t*=180 s, (D) equilibrium modulus, (E) instantaneous modulus and (F) viscosity. Data pooled from two independent experiments. (**: *p*<0.01, ***: *p*<0.001 Mann–Whitney *U* test; see Table 1 for *n* values).

To test whether the expression of LifeAct-GFP and actin-GFP influence viscoelastic cell mechanics, the temporal change in aspirated length was quantified from brightfield microscopy images and used to calculate the instantaneous and equilibrium moduli and viscosity based on the standard linear solid (SLS) model. These well-established parameters approximate cellular mechanical behaviour and provide a useful means of quantitative comparison. Fig. 3A shows the mean aspirated length versus time indicating that cells exhibit characteristic viscoelastic solid-like behaviour with a rapid increase in aspirated length which equilibrates over time. However, for cells expressing actin-GFP, the temporal change in aspirated length did not provide as reliable a fit to the viscoelastic standard linear solid (SLS) model as indicated by significantly lower *R*^2^ values compared to non-transfected cells or those expressing LifeAct-GFP (Fig. 3B). The number of cells analysed in each group is presented in Table 1. Non-transfected hMSCs exhibited equilibrium and instantaneous moduli with median values of 0.15 kPa and 0.96 kPa respectively, similar to values reported in previous studies (Tan et al., 2008, Yu et al., 2010). For cells transfected with LifeAct-GFP there were no significant differences from non-transfected control cells in terms of the aspiration length at 180 s (*p*=0.907), the equilibrium modulus (*p*=0.535), the instantaneous modulus (*p*=0.990) and the viscosity (*p*=0.320; Fig. 3C–F). However this was not the case for hMSCs transfected with actin-GFP which exhibited a significantly shorter aspiration length at 180 s (*p*<0.01; Fig. 3C), which was associated with a significantly higher equilibrium modulus (*p*<0.01; Fig. 3D) compared to controls. Interestingly, the instantaneous modulus and viscosity were unaffected by actin-GFP expression, possibly as this behaviour was dominated by the rapid expansion of the actin-free membrane bleb.

**Fig. 4:**
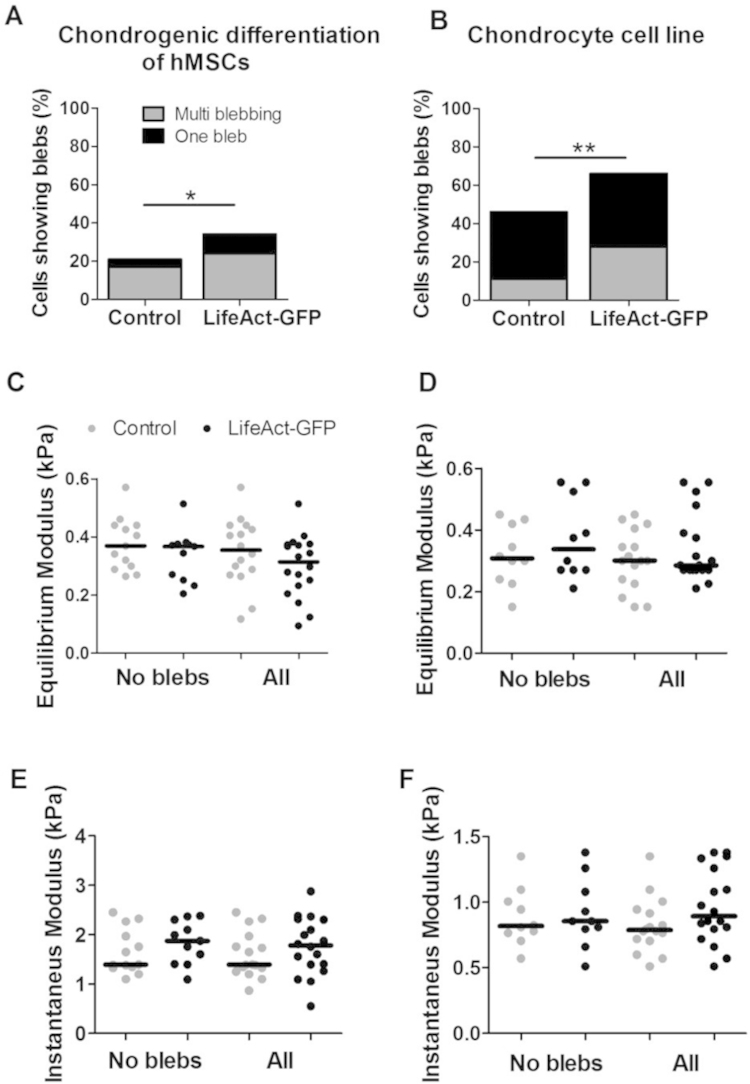
LifeAct-GFP influences membrane blebbing but has no effect on cellular/actin mechanical properties independent of blebbing. Data shown for chondrogenically differentiated hMSCs (A, C and E) and a murine chondrocyte cell line (B, D and F). Histograms showing a significant increase in the percentage of cells exhibiting membrane blebs in cells expressing LifeAct-GFP versus non transfected control cells (A and B; Chi-squared test, *: *p*<0.05, **: *p*<0.001). There were no statistically significant differences between LifeAct-GFP transfected and control cells in terms of the equilibrium modulus (C and D) and the instantaneous modulus (E and F) either for the non blebbing cell fraction or for the entire sample population; Mann–Whitney *U* test. For non blebbing differentiated hMSCs *n*=13 (control), *n*=11 (LifeAct-GFP); chondrocyte cell line *n*=10 (control) and *n*=10 (LifeAct-GFP). See Table 1 for *n* values for all cells.

In the absence of bleb formation, cellular mechanical properties are strongly governed by the actin cortex (Brugues et al., 2010, Haase and Pelling, 2013). Since hMSCs exhibit aspiration-induced membrane blebbing, it was not possible to determine if LifeAct-GFP influenced actin cortex mechanics. We have previously demonstrated that bleb incidence reduces with chondrogenic differentiation of hMSCs as ERM linker protein levels increase (Sliogeryte et al., 2014). Therefore additional studies were performed using both hMSCs differentiated to the chondrogenic lineage and a murine chondrocyte cell line. In these two cell types, blebbing occurred in only 20% and 45% of cells respectively. Interestingly expression of LifeAct-GFP significantly increased membrane blebbing incidence in both cell types (*p*<0.05; Fig. 4A and B) as observed in hMSCs (Fig. 2C). This may be due to competition for actin binding sites between LifeAct-GFP and ERM membrane-actin cortex linker proteins resulting in a weakened membrane–actin cortex bond. To test whether LifeAct-GFP influences actin cortex mechanical properties, the equilibrium and instantaneous moduli were estimated for non-blebbing cell populations. For both chondrogenically differentiated hMSCs and chondrocytes, expression of LifeAct-GFP did not induce any statistically significant differences from non-transfected controls in terms of cellular equilibrium and instantaneous moduli (Fig. 4C–F). This indicates that LifeAct-GFP does not influence the effective mechanical properties of either the actin cortex or the whole cell. The moduli values obtained for these cell types were in broad agreement with previously published values for hMSCs (Tan et al., 2008) and chondrocytes (Trickey et al., 2000). Consistent with findings using cytocompression (Ofek et al., 2009), MSCs exhibited lower equilibrium and instantaneous moduli than chondrocytes.

Our findings expand on previously reported studies demonstrating that LifeAct-GFP does not interfere with actin dynamics (Lemieux et al., 2014, Riedl et al., 2008, Riedl et al., 2010), although the propensity for bleb formation was increased. Robust overexpression of LifeAct-GFP in *Drosophila* nurse cells has been observed to cause severe actin defects including breakdown of cortical actin (Spracklen et al., 2014). It is possible that LifeAct-GFP may block the binding of various proteins including ERM proteins as discussed above, and at very high expression levels may impair actin functionality. No impairment of actin functionality was observed at the level of expression induced in these studies. F-actin distortion, remodelling and the growth of a new actin cortex following mechanically induced bleb formation can be effectively observed with minimal artefacts.

In conclusion, we show that LifeAct provides a versatile and valuable marker for the labelling of F-actin in living cells without disrupting the effective biophysical properties of the actin cortex or the whole cell, although the susceptibility to membrane bleb formation is increased. In contrast, cells transfected with actin-GFP show altered viscoelastic deformation with reduced bleb formation such that cells appear stiffer as quantified by higher equilibrium moduli compared to non-transfected controls.

## Conflict of interest statement

None of the authors have any competing financial interests related to this paper.

## Figures and Tables

**Table 1:** Numbers of cells used for micropipette aspiration experiments. Values in parentheses indicate the percentage of the total number of tested cells that passed the two exclusion criteria.

**Condition**	**Total cells**	**Successful aspirations**	**Used for model *R*^2^>0.95**
**hMSCs**			
Control	68 (100%)	67 (99%)	52 (76%)
LifeAct-GFP	40 (100%)	38 (95%)	28 (70%)
Actin-GFP	40 (100%)	39 (98%)	22 (55%)
**Chondrogenically differentiated hMSCs**			
Control	23 (100%)	22 (96%)	16 (70%)
LifeAct-GFP	41 (100%)	38 (93%)	18 (44%)
**Chondrocyte cell line**			
Control	28 (100%)	27 (96%)	16 (57%)
LifeAct-GFP	36 (100%)	33 (92%)	18 (50%)

**Movie S1:**
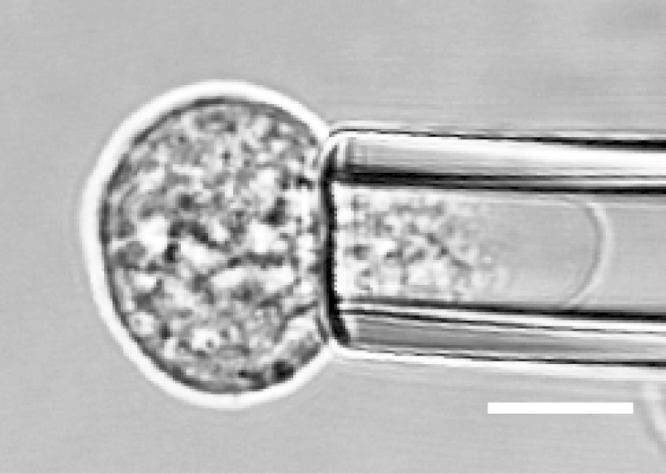
This brightfield movie presents a control cell exhibiting a single bleb in which the membrane detaches from the cortex followed by membrane expansion. Scale bar: 10 µm. A video clip is available online.

**Movie S2:**
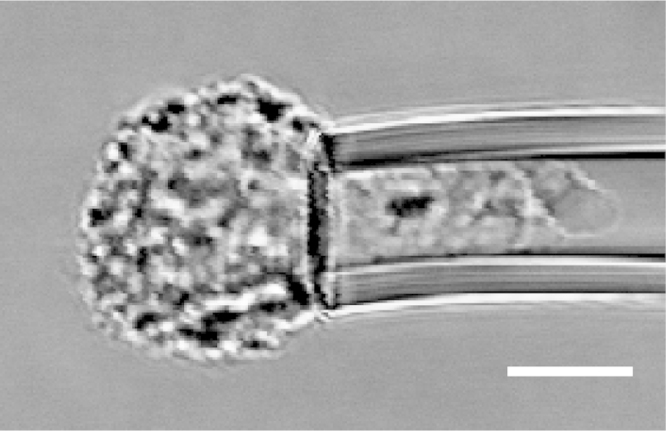
This brightfield movie presents a LifeAct-GFP positive cell with multi blebbing behaviour during aspiration into the micropipette. The single bleb is followed by multiple additional membrane detachments. Scale bar: 10 µm.

**Movie S3:**
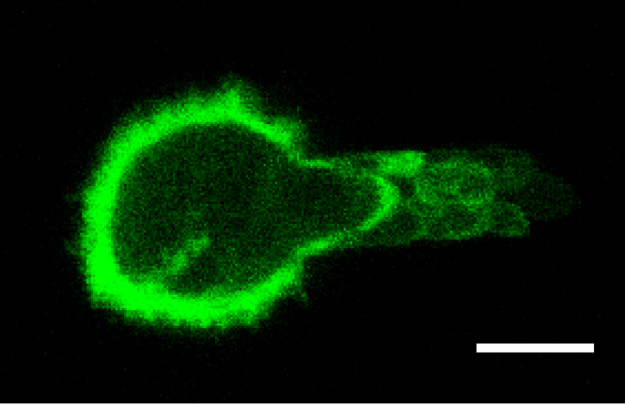
This fluorescent movie presents the same LifeAct-GFP positive cell as in supplementary movie 2 with the fluorescent channel to visualise actin remodelling during micropipette aspiration. Scale bar: 10 µm.

**Movie S4:**
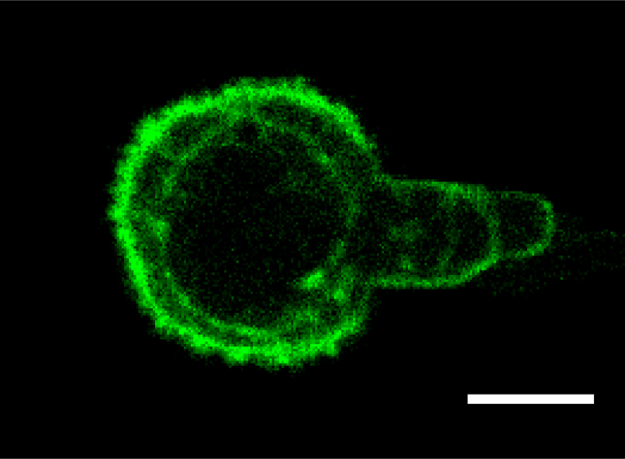
This fluorescent movie presents a LifeAct-GFP positive cell demonstrating multi blebbing behaviour. Scale bar: 10 µm.

**Movie S5:**
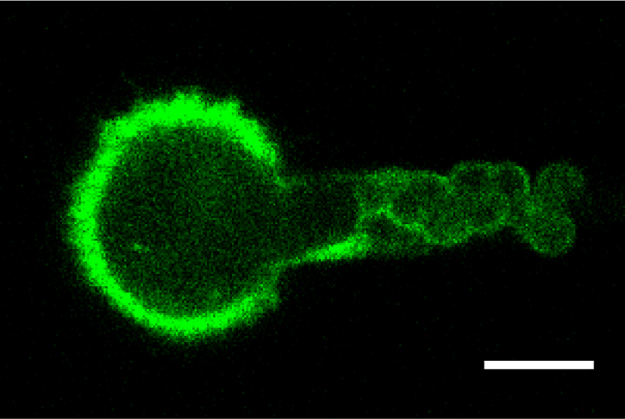
This fluorescent movie presents a LifeAct-GFP positive cell demonstrating multi blebbing behaviour. Scale bar: 10 µm.

**Movie S6:**
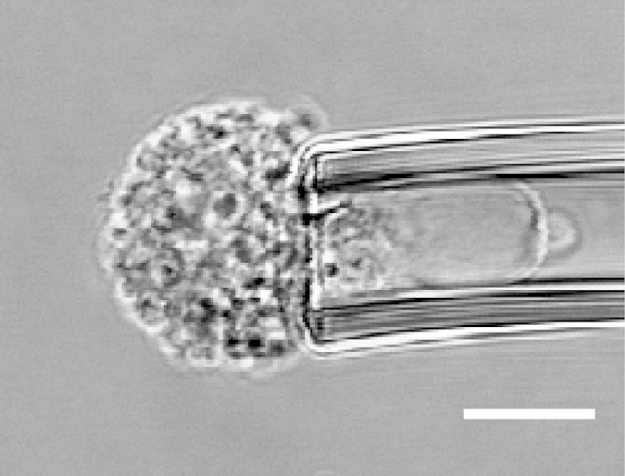
This brightfield movie presents an actin-GFP positive cell exhibiting a single bleb. Scale bar: 10 µm.

**Movie S7:**
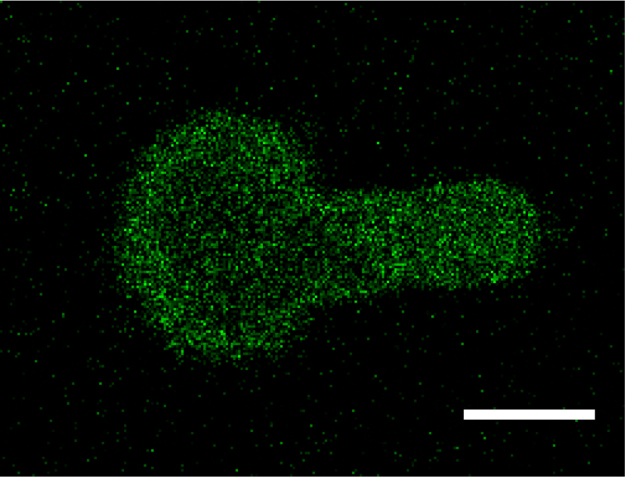
This fluorescent movie presents the same actin-GFP positive cell as in Supplementary movie S6 exhibiting a single bleb. The fluorescent channel allows visualisation of actin remodelling during micropipette aspiration. Scale bar: 10 µm.

## References

[bib1] Aizawa H., Sameshima M., Yahara I. (1997). A green fluorescent protein-actin fusion protein dominantly inhibits cytokinesis, cell spreading, and locomotion in Dictyostelium. Cell Struct. Funct..

[bib2] Belin B.J., Goins L.M., Mullins R.D. (2014). Comparative analysis of tools for live cell imaging of actin network architecture. Bioarchitecture.

[bib3] Brugues J., Maugis B., Casademunt J., Nassoy P., Amblard F., Sens P. (2010). Dynamical organization of the cytoskeletal cortex probed by micropipette aspiration. Proc. Natl. Acad. Sci. USA.

[bib4] Burkel B.M., von Dassow G., Bement W.M. (2007). Versatile fluorescent probes for actin filaments based on the actin-binding domain of utrophin. Cell Motil. Cytoskelet..

[bib5] Charras G.T., Hu C.K., Coughlin M., Mitchison T.J. (2006). Reassembly of contractile actin cortex in cell blebs. J. Cell Biol..

[bib6] Deibler M., Spatz J.P., Kemkemer R. (2011). Actin fusion proteins alter the dynamics of mechanically induced cytoskeleton rearrangement. PLoS One.

[bib7] Endlich N., Otey C.A., Kriz W., Endlich K. (2007). Movement of stress fibers away from focal adhesions identifies focal adhesions as sites of stress fiber assembly in stationary cells. Cell Motil. Cytoskelet..

[bib8] Engelke H., Heinrich D., Rädler J.O. (2010). Probing GFP-actin diffusion in living cells using fluorescence correlation spectroscopy. Phys. Biol..

[bib9] Fackler O.T., Grosse R. (2008). Cell motility through plasma membrane blebbing. J. Cell Biol..

[bib10] Feng Z., Ning Chen W., Vee Sin Lee P., Liao K., Chan V. (2005). The influence of GFP-actin expression on the adhesion dynamics of HepG2 cells on a model extracellular matrix. Biomaterials.

[bib11] Haase K., Pelling A.E. (2013). The role of the actin cortex in maintaining cell shape. Commun. Integr. Biol..

[bib12] Ingber D.E. (2006). Mechanical control of tissue morphogenesis during embryological development. Int. J. Dev. Biol..

[bib13] Janmey P.A., Weitz D.A. (2004). Dealing with mechanics: mechanisms of force transduction in cells. Trends Biochem. Sci..

[bib14] Johnson H.W., Schell M.J. (2009). Neuronal IP3 3-kinase is an F-actin-bundling protein: role in dendritic targeting and regulation of spine morphology. Mol. Biol. Cell.

[bib15] Lee C.W., Vitriol E.A., Shim S., Wise A.L., Velayutham R.P., Zheng J.Q. (2013). Dynamic localization of G-actin during membrane protrusion in neuronal motility. Curr. Biol..

[bib16] Lemieux M.G., Janzen D., Hwang R., Roldan J., Jarchum I., Knecht D.A. (2014). Visualization of the actin cytoskeleton: different F‐actin‐binding probes tell different stories. Cytoskeleton.

[bib17] Lukinavicius G., Reymond L., D׳Este E., Masharina A., Gottfert F., Ta H., Guther A., Fournier M., Rizzo S., Waldmann H., Blaukopf C., Sommer C., Gerlich D.W., Arndt H.D., Hell S.W., Johnsson K. (2014). Fluorogenic probes for live-cell imaging of the cytoskeleton. Nat. Methods.

[bib18] Ofek G., Willard V.P., Koay E.J., Hu J.C., Lin P., Athanasiou K.A. (2009). Mechanical characterization of differentiated human embryonic stem cells. J. Biomech. Eng..

[bib19] Pattappa G., Thorpe S.D., Jegard N.C., Heywood H.K., de Bruijn J.D., Lee D.A. (2013). Continuous and uninterrupted oxygen tension influences the colony formation and oxidative metabolism of human mesenchymal stem cells. Tissue Eng. Part C Methods.

[bib20] Pollard T.D., Cooper J.A. (2009). Actin, a central player in cell shape and movement. Science.

[bib21] Pravincumar P., Bader D.L., Knight M.M. (2012). Viscoelastic cell mechanics and actin remodelling are dependent on the rate of applied pressure. PLoS One.

[bib22] Riedl J., Flynn K.C., Raducanu A., Gartner F., Beck G., Bosl M., Bradke F., Massberg S., Aszodi A., Sixt M., Wedlich-Soldner R. (2010). Lifeact mice for studying F-actin dynamics. Nat. Methods.

[bib23] Riedl J., Crevenna A.H., Kessenbrock K., Yu J.H., Neukirchen D., Bista M., Bradke F., Jenne D., Holak T.A., Werb Z., Sixt M., Wedlich-Soldner R. (2008). Lifeact: a versatile marker to visualize F-actin. Nat. Methods.

[bib24] Sato M., Theret D.P., Wheeler L.T., Ohshima N., Nerem R.M. (1990). Application of the micropipette technique to the measurement of cultured porcine aortic endothelial cell viscoelastic properties. J. Biomech. Eng..

[bib25] Sliogeryte K., Thorpe S.D., Lee D.A., Botto L., Knight M.M. (2014). Stem cell differentiation increases membrane-actin adhesion regulating cell blebability, migration and mechanics. Sci. Rep..

[bib26] Spracklen A.J., Fagan T.N., Lovander K.E., Tootle T.L. (2014). The pros and cons of common actin labeling tools for visualizing actin dynamics during Drosophila oogenesis. Dev. Biol..

[bib27] Tan S.C., Pan W.X., Ma G., Cai N., Leong K.W., Liao K. (2008). Viscoelastic behaviour of human mesenchymal stem cells. BMC Cell Biol..

[bib28] Theret D.P., Levesque M.J., Sato M., Nerem R.M., Wheeler L.T. (1988). The application of a homogeneous half-space model in the analysis of endothelial cell micropipette measurements. J. Biomech. Eng..

[bib29] Thompson C.L., Chapple J.P., Knight M.M. (2014). Primary cilia disassembly down-regulates mechanosensitive hedgehog signalling: a feedback mechanism controlling ADAMTS-5 expression in chondrocytes. Osteoarthr. Cartil..

[bib30] Titushkin I., Cho M. (2011). Altered osteogenic commitment of human mesenchymal stem cells by ERM protein-dependent modulation of cellular biomechanics. J. Biomech..

[bib31] Trickey W.R., Lee G.M., Guilak F. (2000). Viscoelastic properties of chondrocytes from normal and osteoarthritic human cartilage. J. Orthop. Res. – Off. Publ. Orthop. Res. Soc..

[bib32] Wann A.K.T., Zuo N., Haycraft C.J., Jensen C.G., Poole C.A., McGlashan S.R., Knight M.M. (2012). Primary cilia mediate mechanotransduction through control of ATP-induced Ca^2+^ signaling in compressed chondrocytes. Faseb J..

[bib33] Yourek G., Hussain M.A., Mao J.J. (2007). Cytoskeletal changes of mesenchymal stem cells during differentiation. ASAIO J..

[bib34] Yu H., Tay C.Y., Leong W.S., Tan S.C., Liao K., Tan L.P. (2010). Mechanical behavior of human mesenchymal stem cells during adipogenic and osteogenic differentiation. Biochem. Biophys. Res. Commun..

[bib35] Zhelev D.V., Needham D., Hochmuth R.M. (1994). Role of the membrane cortex in neutrophil deformation in small pipets. Biophys. J..

